# Single-dose baclofen-induced neurotoxicity in a patient with end stage renal disease: case report

**DOI:** 10.1186/s12882-018-1167-z

**Published:** 2018-12-11

**Authors:** Emad Khazneh, Alaa Shamlawi, Kamel Jebrin, Zakaria Hamdan, Osama Sawalmeh

**Affiliations:** 1 Nephrology Consultant, Nephrology department, An-Najah National University Hospital, Nablus, Palestine; 2 Internal Medicine resident, Internal Medicine Department, An-Najah National University Hospital, Nablus, Palestine; 3 Intern Doctor, Internal Medicine Department, An-Najah National University Hospital, Nablus, Palestine

**Keywords:** Baclofen, Toxicity, Neurotoxicity, End stage renal disease, ESRD, Chronic kidney disease, Overdose, Case report

## Abstract

**Background:**

Baclofen is a centrally acting GABA_B_ receptor agonist and it is used widely for the treatment of spasticity, persistent hiccups and multiple sclerosis. The renal system is the main route of excretion, thus people with suboptimal renal function are prone to baclofen intoxication. Multiple doses of baclofen have been associated with toxicity, but it is very unusual that single dose can do so.

**Case presentation:**

A 47 year old female patient with end stage renal disease (ESRD) presented with a sudden onset of altered mental status and state of unconsciousness after the ingestion of one tablet of baclofen 25 mg. All other possible causes were ruled out and a diagnosis of baclofen toxicity was considered. The patient showed dramatic improvement after an extra two sessions of hemodialysis.

**Conclusions:**

We highly recommend that more educational efforts are made for health care professionals about the possible risk of baclofen toxicity among kidney-impaired patients. We also recommend avoiding baclofen use if evidence of chronic renal disease is present and to seek other alternatives for pain management.

## Background

Baclofen, β-4-chlorophenyl gamma-aminobutyric acid, is a natural derivative of the neurotransmitter gamma-aminobutyric acid (GABA) [1, 2]. Specifically, it is considered as an active agonist for GABA_B_ receptors [2]. Baclofen acts centrally, thus it has been used to treat skeletal muscle spasms, persistent hiccups, multiple sclerosis and other spinal cord lesion-induced spasticity [1, 3, 4, 5]. Baclofen is absorbed primarily by the gastrointestinal tract and more than 80% is excreted by the kidneys [6]. This explains why people with reduced kidney function are at higher risk of intoxication than those with normal kidney function. The half-life of Baclofen ranges between 4.5–6.8 h. This half-life increases in patients with impaired renal function [7]. Baclofen can cross the Blood-Brain barrier only very slowly for central nervous system penetration but if the half-life is extended this leads to more CNS penetration and its effect in term of CNS depression can be easily pronounced [3, 6].

This case report presents a patient with end-stage renal disease suffering from single-dose induced Baclofen toxicity. She presented with altered mental status and improved dramatically after high-efficiency intensive hemodialysis sessions.

## Case presentation

A 47-year-old female patient presented to the dialysis unit with decreased level of consciousness and sleepiness of two days duration. Her past medical history is end stage renal disease and regular hemodialysis 3 times/week for 2.5 years, controlled hypertension. She is non-diabetic. Her past surgical history included arteriovenous fistula before 2.5 years, appendectomy and tonsillectomy long time ago.

The patient was in her usual state of health of moderate exercise tolerance until two days before admission when she started to experience lower back and bilateral knee pain that was vague in nature and associated with insomnia. That time, she received one tablet of baclofen 25 mg from her sister. Her sister used to ingest baclofen for chronic neck pain. The patient fell into a deep sleep throughout that night and entire next day without any wakefulness periods. Two days later, she went to her usual hemodialysis session and there the medical personnel noticed her high blood pressure and a state of unconsciousness for which she was sent back to the emergency department after the hemodialysis session. The family denied any previous similar episodes or limb weakness, numbness, dysarthria, dysphagia or mouth deviation before the event. There is no history of fever, photophobia, neck stiffness, falling down or any psychosocial problems. She had not travelled or had any contact with sick people. She was compliant to her hemodialysis sessions. She is a 20 pack-year smoker but does not use any illegal drugs or consume alcoholic drinks. Her home medications were: Atenolol 50 mg/day, Amlodipine 5 mg/ day, CaCO_3_ 600 mg /day and alfacalcidol 0.25 ugm/day.

On examination, the vital signs were as follows: Temperature: 36.4 °C, Blood pressure: 220/110, pulse: 95 beat/minute, respiratory rate: 14/min, oxygen saturation: 95% on room air. On admission, the Glasgow Coma Scale was 9/15. She was unconscious. Deep tendon reflexes were absent. There was no obvious facial asymmetry, left pupil was round and reactive to light (right eye is artificial due to previous trauma). Gag reflex was intact but the cranial nerves could not be assessed. The fundus was examined and no papilledema or hemorrhage were seen. The breathing sounds were heard bilaterally on the chest with no added sounds. Abdominal examination was unremarkable.

Lab results at admission were: Hemoglobin: 16.1 g/dL, White blood cells: 6.8 K/ul, platelets: 155 K/ul, Sodium: 138 mEq/L, potassium: 4.4 mEq/L, chloride: 95 mEq/L, CRP: 1.6 mg/L, Glucose: 99 mg/dL, ABG’s: pH:7.44, pCO_2_: 36.6 mmHg, pO_2_: 88 mmHg, HCO_3_: 24.4 mEq/L, albumin: 4.4 g/dL, alkaline phosphatase:88, total bilirubin: 0.5 mg/dL, direct bilirubin: 0.2 mg/dL, BUN: 27 mg/dL, Creatinine: 5.5 mg/dL. Liver function tests were within normal ranges. To rule out any brain ischemia or hemorrhage, brain computed topography (CT scan) was done on the day of admission and Magnetic resonance imaging (MRI) of the brain was done on the second day of admission. CT scan showed no significant lesions, no hemorrhage or any other defects. MRI showed no signs of recent ischemic stroke, intracranial hemorrhage, space-occupying lesion or midline shift. The hypertension was controlled by labetalol IV infusion pump.

After ruling out all possible causes of her decreased level of consciousness, including Posterior reversible encephalopathy syndrome (PRES), baclofen toxicity was considered as the cause. She was started on flumazenil 0.25 mg and on intensive daily ultrafiltration hemodialysis sessions since the first day of admission with total of three extra hemodialysis sessions. The improvement started to be noticed at the second day when she was semi-conscious, hallucinating, obeying commands as ordered but still not oriented to time, place or person. GCS was 10/15. The dramatic improvement was on the next day after a total of two extra hemodialysis sessions, she was fully awake, GCS 15/15, able to obey commands but still not well oriented so a third extra session was considered. After three days of admission and a total of five hemodialysis sessions, she was discharged after she had returned to her previous baseline state of health and she was instructed not to receive baclofen again.

## Discussion

Baclofen is a natural derivative agonist of GABA_B_ receptors at the level of the spinal cord thus producing its attenuating effect on the muscle tone if used within the therapeutic range [1]. The daily therapeutic dose ranges between 5 and 60 mg [7, 8]. Adverse effects with baclofen overdose have been noticed among people with normal kidney function such as sedation, confusion, muscle weakness and impaired consciousness [1] and these effects are attributed to its inhibitory effect on the central nervous system [6]. The reported doses of intoxication were between 80 mg and 2500 mg with the more severe among those who have ingested more than 200 mg requiring more intensive care unit admissions and for longer durations [9].

The situation becomes more complicated in those with impaired renal function as baclofen is mainly excreted by the renal system (85–90%) and the rest, 10–15%, becomes metabolized by the liver [6]. Based on this data, and knowing that the liver function is intact, or, at least, not impaired, as the liver enzymes and other related laboratory variables were within the normal range, the impaired kidney functioning capacity is the only one to be blamed as the cause of baclofen toxicity. The reported doses of baclofen toxicity among patients with chronic kidney disease ranges between 5 mg and 60 mg per day, and most of the patients were known to have advanced kidney disease [10]. The onset of symptoms usually started 2–3 days after the ingestion of baclofen [4, 10] but as early as 24 h have also been documented among those with end-stage renal disease [6]. However, our case developed a state of sleepiness and unconsciousness as soon as 12 h post ingestion of a single dose of 25 mg. The impaired excretion is responsible for the toxic effects of baclofen even with normal therapeutic doses as it accumulates and induces neurotoxicity [4].

Among the presumed differentials is PRES, posterior reversible encephalopathy syndrome. PRES is a group of neurological signs and symptoms and characteristic radiologic findings that are usually reversible [11]. Clinically, PRES is diagnosed by acute onset of headache, seizures, visual problems and encephalopathy^,^ in addition to the radiologic findings of vasogenic edema, either by CT or MRI (Preferred), mainly affecting the parieto-occipital lobes [12]. Referring to our patient, PRES could easily be excluded as, by definition, PRES should have radiologic findings as part of the diagnostic criteria and both CT and MRI were done for the patient and no one could identify any consistent findings for PRES or any other intracranial lesion that might explain the clinical picture of the patient.

The manifestations of baclofen toxicity are dizziness, nausea, vomiting, respiratory depression, altered mental status, ataxia, dystonia and coma [3, 6, 8, 13]. Reversible akinetic mutism [14] and severe comatose state with absent brain stem reflexes have been documented with severe baclofen intoxication [15]. The degree of chronic renal failure varied among patients from acute kidney injury to stage 5 chronic renal failure and eventually end stage renal disease [6, 15].

The mainstay of treatment is supportive and close monitoring for possible respiratory compromise [6]. Hemodialysis is also a very reliable method of treatment as it decreases baclofen half-life in ESRD patients from 15 to 2 h [16]. Some reported cases improved dramatically after only one session as it is thought to eliminate baclofen from the body as do normal kidneys [16–18] but other patients needed up to five sessions for complete recovery [6]. Our patient started to show improvement after two hemodialysis sessions and the complete recovery was gained after a total of five. Continuous Renal Replacement Therapy seems to be a good substitution for those patients who are not a candidate for hemodialysis [6].

In the published literature, there is still a much debate of what dose of baclofen to use in patients with impaired kidney function and when to start decreasing or discontinuing the dose [4, 7, 15]. Roberts et al. [6] provided a clear suggestion of totally avoiding baclofen use among ESRD and acute renal failure patients and those with a glomerular filtration rate less than 60 ml/minute. They also suggested to use the lowest possible dose for those with mild chronic kidney disease, GFR more than 60 ml/minute [6].

Another presumed hypothesis to explain what happened to the patient is the finding of significantly elevated blood pressure at the time of diagnosis and whether or not it might have contributed to the increased risk of baclofen toxicity, even with a single dose as noticed in our case. Up to the authors’ knowledge, not one study was reported to prove or disprove this hypothesis as most of the reported cases had normal or slightly elevated blood pressures [1, 3, 4, 8, 15].

## Conclusions

In conclusion**,** Baclofen toxicity is a serious, life threatening condition, especially for those with impaired renal function. Patients with suboptimal renal function presenting with altered mental status with a history of baclofen ingestion should prompt the diagnosis of baclofen toxicity. As soon as the diagnosis is made and hemodialysis is initiated, it can be completely reversible without any sequelae. More efforts should be done towards educating health care professionals about the possible risks of baclofen use with ESRD patients. We also recommend avoiding baclofen use among patients with decreased renal function, even if mild chronic renal failure, as the accumulation in the CNS is cumulative and depends on the renal excretion capacity. Therefore, we highly recommend seeking other alternatives.

## Abbreviations

CaCO_3_: calcium bicarbonate
CNS: central nervous system
CT: computed topography
ESRD: end stage renal disease
GABA: gamma-aminobutyric acid
GCS: Glasgow coma scale
MRI: magnetic resonance imaging
